# Development, validation and item reduction of a food literacy questionnaire (IFLQ-19) with Australian adults

**DOI:** 10.1186/s12966-022-01351-8

**Published:** 2022-09-01

**Authors:** Courtney Thompson, Rebecca Byrne, Jean Adams, Helen Anna Vidgen

**Affiliations:** 1 Queensland University of Technology (QUT), Faculty of Health, School of Exercise and Nutrition Sciences, Victoria Park Road, Kelvin Grove, QLD 4059 Australia; 2 Queensland University of Technology (QUT), Faculty of Health, School of Exercise and Nutrition Sciences, Centre for Children’s Health Research (CCHR), Graham Street, South Brisbane, QLD 4101 Australia; 3 Centre for Diet and Activity Research (CEDAR), MRC Epidemiology Unit, University of Cambridge School of Clinical Medicine, Cambridge, UK

**Keywords:** Food literacy, Survey, Item response theory, Rasch measurement, Partial credit model, Test–retest reliability, Validity

## Abstract

**Background:**

Food literacy is theorised to improve diet quality, nutrition behaviours, social connectedness and food security. The definition and conceptualisation by Vidgen & Gallegos, consisting of 11 theoretical components within the four domains of planning and managing, selecting, preparing and eating, is currently the most highly cited framework. However, a valid and reliable questionnaire is needed to comprehensively measure this conceptualisation. Therefore, this study draws on existing item pools to develop a comprehensive food literacy questionnaire using item response theory.

**Methods:**

Five hundred Australian adults were recruited in Study 1 to refine a food literacy item pool using principal component analysis (PCA) and item response theory (IRT) which involved detailed item analysis on targeting, responsiveness, validity and reliability. Another 500 participants were recruited in Study 2 to replicate item analysis on validity and reliability on the refined item pool, and 250 of these participants re-completed the food literacy questionnaire to determine its test–retest reliability.

**Results:**

The PCA saw the 171-item pool reduced to 100-items across 19 statistical components of food literacy. After the thresholds of 26 items were combined, responses to the food literacy questionnaire had ordered thresholds (targeting), acceptable item locations (< -0.01 to + 1.53) and appropriateness of the measurement model (*n* = 92% expected responses) (responsiveness), met outfit mean-squares MSQ (0.48—1.42) (validity) and had high person, item separation (> 0.99) and test–retest (ICC 2,1 0.55–0.88) scores (reliability).

**Conclusions:**

We developed a 100-item food literacy questionnaire, the IFLQ-19 to comprehensively address the Vidgen & Gallegos theoretical domains and components with good targeting, responsiveness, reliability and validity in a diverse sample of Australian adults.

**Supplementary Information:**

The online version contains supplementary material available at 10.1186/s12966-022-01351-8.

## Background

**Fig. 1:**
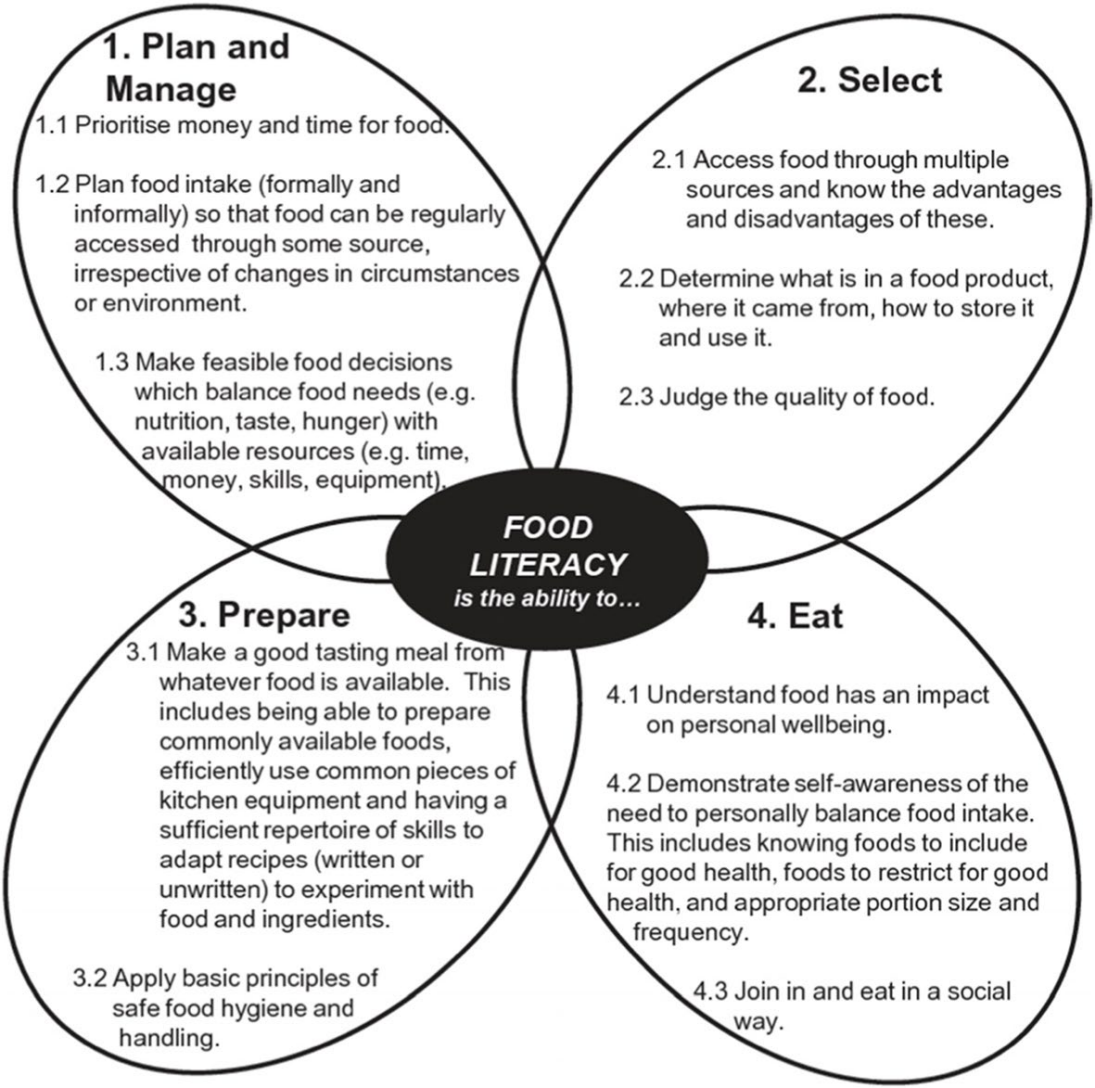
Domains and components of food literacy by Vidgen & Gallegos [1, 2]

Vidgen & Gallegos [1] define food literacy as “… a collection of inter-related knowledge, skills and behaviours required to plan, manage, select, prepare and eat food to meet needs and determine intake” and the “… scaffolding that empowers individuals, households, communities or nations to protect diet quality through change and strengthen dietary resilience over time.” Their conceptualisation consists of 11 components organised under four inter-related domains of planning and managing, selecting, preparing and eating (see Fig. 1).

Recently, an international consensus study [3] and a scoping review of 549 publications on food literacy [4] found that there is agreement internationally with the Vidgen & Gallegos [1] definition and conceptualisation of food literacy. This definition is the first to be empirically derived, and is proposed by researchers as the most current, predominant approach to food literacy that is able to significantly advance the field [5–7].

While food literacy surveys have been developed [8–19], these tend to be the result of expert consensus on food literacy rather than consultation with the general public. However, the Vidgen & Gallegos [1] conceptualisation was derived from an analysis of knowledge, skills and behaviours used by the general public to protect their diet quality through change. Where papers stated use of the Vidgen & Gallegos model [1] to guide tool development, analysis of these tools indicated that not all components in the model are measured; and thus, the domains or construct of food literacy are not comprehensively captured [20]. Being able to discernibly measure each component is likely to be more useful in guiding policy and practice than domain or overall scores which mask important differences across the spectrum of food literacy between individuals.

In order to address the limitations with existing surveys, work has been conducted by Vidgen and colleagues since 2014 to develop a comprehensive food literacy measure. This has included a four-part content validity study involving, 1) Expert feedback on the construct of food literacy and its components [2]; 2) A review of these constructs against interventions [2]; 3) Young people’s feedback on the construct [2] and; 4) An international item pool consensus study [3, 20]. Questionnaire development was further informed by reflections on what a measure needs to encompass [21, 22]. A face validity study using cognitive interviews was then conducted with a purposefully selected sample of the general public that was representative of demographic characteristics identified in earlier research [3, 23]. This series of studies generated a 171-item pool that reflected the 11 components proposed by Vidgen & Gallegos [1] (see Fig. 1). However, the reliability, validity and consistency of this item pool had not been assessed.

In existing food literacy surveys, psychometric properties are typically assessed using Classical Test Theory (CTT) methods. While CTT is widely used [24], it carries a number of assumptions that limit scale development and generalisability of findings [25–27].

Item Response Theory (IRT) offers one alternative to CTT. It has been defined as, “…a mathematical model that relates a test-taker’s latent trait or ability score with the probability of responding in a specific response category of an item” [28]. It consists of a family of models which are not bound by the same assumptions as CTT. In particular, IRT produces item-level information rather than scale-level information which overcomes a major limitation imposed by CTT. This is the assumption that all items contribute equally to the total questionnaire score; thus IRT is more nuanced, allowing individuals to be higher on one construct and lower on another [25]. IRT is increasingly used in public health research [29–34] and more specifically, in public health nutrition research [35, 36].

The purpose of this paper is to describe methods used to develop a food literacy questionnaire which comprehensively measures the four domains and 11 components of food literacy. This involved detailed item analysis on, 1) targeting: the extent to which the distribution in the sample matches the range measured by the scale [37]; 2) responsiveness: the ability of an instrument to detect change accurately when it has occurred [38]; 3) reliability: the extent to which results obtained by a scale can be replicated and scores are free from random error [39]; and 4) validity: the extent to which a scale measures what is intended, and is free of systematic error [3, 23, 39]. This aimed to develop a refined item pool (Study 1), replicate item analysis on validity and reliability on the refined item pool from Study 1 (Study 2); and determine test–retest reliability of the refined item pool from Study 1 (Study 3).

## Methods

### Study 1

#### Study participants and recruitment

This study purposefully sampled a diverse range of residents of Australia, over 18 years of age: participant quotas can be seen in Table 1 based on Australian census data [40, 41]. Participants were recruited via double-opt-in market research panels through Qualtrics [42] and approached via email, in-app or SMS notifications to participate in an online survey between the 25^th^ November 2020 and 14^th^ December 2020. A sample size of 500 participants achieved the upper boundary of recommended sample sizes [43–45]. If participants were less than 18 years of age or did not agree to participate, they were screened out.

**Table 1:** Demographic characteristics of study participants

Categories (Qualtrics quotas, %)	Study 1, *n* = 504 (%)	Study 2, *n* = 503 (%)	Study 3, *n* = 269 (%)
**Gender**			
Male (51)	243 (48)	266 (53)	156 (58)
Female (49)	256 (51)	237 (47)	113 (42)
Not disclosed	5 (1)	0 (0)	0 (0)
**Age**			
18–24 (16)	78 (15)	43 (9)	26 (10)
25–34 (18)	91 (18)	99 (20)	50 (18)
35–44 (17)	89 (17)	93 (18)	42 (16)
45–54 (16)	76 (15)	88 (17)	44 (16)
55–64 (15)	74 (15)	81 (16)	45 (17)
65–74 (19)	69 (14)	74 (15)	44 (16)
75–84	25 (5)	23 (4)	17 (6)
85 +	2 (1)	2 (1)	1 (1)
**Annual individual income**^a^			
$0-$15,599 (19)	89 (18)	94 (19)	39 (15)
$15,600–25,999 (15)	70 (14)	62 (12)	41 (15)
$26,000-$41,599 (16)	78 (15)	87 (17)	55 (20)
$41,600-$64,999 (16)	89 (18)	80 (16)	43 (16)
$65,000-$90,999 (18)	78 (15)	83 (17)	44 (16)
$91,000-$155,999 (16)	70 (14)	77 (15)	40 (15)
$156,000 +	30 (6)	20 (4)	07 (3)
**Ancestry**^b^			
Oceanian	362 (58)	372 (63)	193 (62)
North-West European	147 (24)	104 (17)	58 (18)
Southern and Eastern European	29 (4)	34 (6)	18 (6)
North African and Middle Eastern	7 (1)	9 (1)	40 (1)
South-East Asian	15 (2)	12 (2)	70 (2)
North-East Asian	37 (6)	28 (4)	190 (6)
Southern and Central Asian	11 (2)	23 (4)	90 (3)
Peoples of the Americas	2 (1)	2 (1)	10 (1)
Sub-Saharan African	1 (1)	4 (1)	20 (1)
Preferred not to answer	5 (1)	3 (1)	0 (0)
**State**			
Queensland	96 (19)	96 (19)	51 (19)
New South Wales	136 (27)	150 (30)	83 (31)
Victoria	155 (31)	141 (28)	72 (27)
South Australia	51 (10)	41 (8)	19 (7)
Western Australia	41 (8)	41 (8)	260 (9)
Tasmania	16 (3)	29 (6)	130 (5)
Northern Territory	2 (1)	0 (0)	0 (0)
Australian Capital Territory	7 (1)	5 (1)	5 (2)
**Education (highest achieved)**			
Primary school	92 (18)	75 (15)	35 (13)
High school	91 (18)	94 (19)	48 (18)
Trade or other certificate	79 (16)	87 (17)	560 (21)
Diploma	58 (12)	56 (11)	310 (11)
Bachelor degree	131 (26)	145 (29)	780 (29)
Post-graduate degree	53 (10)	46 (9)	210 (8)
**Current employment status**			
Employed	303 (60)	286 (57)	1430 (53)
Not employed	201 (40)	217 (43)	1260 (47)
**Socio-economic advantage and disadvantage**^c^			
1 (most disadvantaged)	41 (8)	51 (10)	270 (10)
2	36 (7)	30 (6)	120 (4)
3	31 (6)	44 (8)	250 (9)
4	50 (10)	39 (8)	210 (8)
5	47 (9)	40 (8)	220 (8)
6	43 (9)	56 (11)	31 (12)
7	41 (8)	41 (8)	21 (8)
8	74 (15)	71 (14)	37 (14)
9	88 (17)	65 (13)	36 (13)
10 (most advantaged)	45 (9)	65 (13)	37 (14)
Could not be categorised	8 (2)	1 (1)	0 (0)
**Remoteness area index**^d^			
Major cities of Australia	377 (69)	391 (68)	224 (71)
Inner regional Australia	113 (21)	134 (23)	69 (22)
Outer regional Australia	45 (8)	43 (7)	16 (5)
Remote Australia	5 (1)	4 (1)	2 (1)
Very remote Australia	3 (1)	3 (1)	1 (1)

^a^Annual individual income brackets were determined by Qualtrics, closest to the Australian Bureau of Statistics (ABS) classification [41]

^b^Australian Bureau of Statistics (ABS) standard classification for cultural and ethnic groups (ASCCEG) [46]

^c^Australian Bureau of Statistics (ABS) postal area code by indexes for Australia (SEIFA) [40]. An area with a high score on this index has a relatively high incidence of advantage and a relatively low incidence of disadvantage. Eight postcodes were not available in the SEIFA database

^d^Australian Bureau of Statistics (ABS) postal area code by remoteness area [47]. Some postcodes covered more than one category

#### Item pool

The 171-item pool was developed from previous research [3, 23] as described above. Each of the 11 components in the Vidgen & Gallegos food literacy model [1] were represented by 10–40 items (see Appendix 1). All items were on a 5-point likert scale of either: strongly disagree to strongly agree, never to always, not at all important to extremely important, not knowledgeable at all to extremely knowledgeable, or not at all sure to very sure. Twenty-seven items were reverse scored.

#### Data collection

Participants were asked to complete the item pool, which Qualtrics estimated would take 29-min. Participants were able to track their progress via a progress bar and to exit and re-enter the questionnaire and continue at any time prior to participant quotas being met. On completion of the food literacy questionnaire, the following demographic data (12 questions) was collected in addition to that obtained during screening: sex, ancestry, postcode, highest level of education obtained, employment status, income, number of people in their household (including children), who is primarily responsible for meal preparation and cooking in the household and how many times per week meals are prepared and cooked at home (see Table 1). Participants received compensation in the form of cash, vouchers or points through Qualtrics in accordance with the length of the questionnaire.

#### Data analysis

Participant responses were downloaded into an Excel spreadsheet [48] and data cleaning was undertaken to remove incomplete respondents. Participants who completed all items in the questionnaire were used in the analysis. Demographic information was summarised using descriptive statistics in Excel, and postcodes were analysed to determine socio-economic advantage or disadvantage using SEIFA [40] and level of remoteness [47].

#### Checking assumptions of methods 

Statistical analyses were undertaken using R Studio, version 1.4.1717 and SPSS, version 27.0.1.0. As the likert-scales all contained five ordinal response categories, and one item had six response categories, the Partial Credit Model (PCM), a type of Item Response Theory (IRT) was chosen for this analysis.

Prior to running the analysis, the assumption of unidimensionality (that participants responses are based on the underlying concept, not previous items) needs to be met [49]. A Principal Components Analysis (PCA) was run using varimax rotation on each of the 11 components of food literacy in SPSS, version 27.0.1.0. First, sampling adequacy and the data’s suitability for reduction were assessed, where a Kaiser-Meyer Olkin (KMO) score of > 0.5 [50] and a significant Bartlett’s test confirmed a PCA was able to be appropriately conducted on the data. The eigenvalues, scree plot and rotated component matrix from the PCA were then assessed for the number of principal components and loadings. To determine a clear principal component structure, items were removed one at a time starting with the item with the lowest loading until all items loading < 0.32 were removed [51]. Then, items were removed one at a time from the highest cross loading until all items loading > 75% were removed [51]. A total variance explained of > 50% indicated adequate principal component structure [50]. Once a clear structure was developed, C.T. reviewed the principal components against the food literacy components and developed a questionnaire structure. Where one principal component did not comprehensively assess the theoretical food literacy component, the second or third principal component was included. Final decisions were discussed with H.A.V. and R.B. until agreement was reached. For food literacy components in which the PCA identified more than one principal component, these were separated and labelled as ‘statistical components’ which were taken forward for future analysis.

The PCM was then run for each statistical component following methods proposed by Wright & Masters [52], using the eRm package [53] and script adapted for R from Wind & Hua [54]. These methods are standard procedures when conducting IRT analysis, with further information available elsewhere [28, 29, 49, 55, 56].

The resultant PCM outputs were reviewed, where thresholds for the statistical tests are described below. However, it should be noted that these thresholds provide guidance for researcher judgements and are not strict criteria.

##### Assessment of targeting

Item thresholds were reviewed to determine if response categories were working as intended. Item thresholds should be sequentially ordered from most negative to most positive values. If they are not ordered, this suggests that participants are not reliability discriminating between response categories [57]. In these cases, disordered response categories were combined with subsequent response categories to meet ordering requirements [58]. This relationship was graphically described using Category Probability Curves (CPCs), with the width of a threshold indicative of the probability of the category response being chosen [59, 60].

To determine if items mapped out a discernible line of increasing intensity across the continuum of food literacy, item locations, the overall item difficulty were extracted [58]. Item locations should span equally from -2 to + 2 [55]; locations < -2 were considered too easy and >  + 2 too difficult and were removed. Further, item locations on the same logit (the item difficulty estimate determined using a simple logistic function) [55, 56] were also considered for removal as they are assessing the same level of food literacy. This was determined using scale-item maps [61].

##### Assessment of responsiveness

To determine if the food literacy items adequately represented respondent’s levels of food literacy, the range of item locations, thresholds and person locations (the person ability estimates) were compared [62]. Item locations and thresholds distributed within ± 1 logits of the person locations were considered consistent with expectations. Floor effects (< -1 logits of the item thresholds) and ceiling effects (> + 1 logits of the item thresholds) were assessed. The upper limit for the proportion of participants with floor or ceiling effects is 15% [63] and 20% respectively [64, 65]. Items with high floor or ceiling effects were considered for removal. This relationship was visualised using person-item maps.

##### Assessment of reliability

Person separation reliability were extracted to determine if the scale was sensitive in distinguishing between low and high performers [52]. Values of > 0.70 were considered as able to discriminate among people with differing levels of food literacy [56]. Item separation reliability were extracted to determine if the person sample was large enough to confirm item difficulty [62, 66]. Values of > 0.70 are indicative of high item separation reliability [56].

##### Assessment of validity

Item fit was analysed to determine if items appropriately measured the food literacy model. Outfit mean-squares (MSQs) between 0.5 and 1.5 were considered indicative of items that were productive for measurement. Values > 2.0 were considered to degrade the measurement and were considered for removal [56, 67].

Person fit was analysed using Z-statistics to identify atypical response patterns, such as participants who randomly selected responses, exaggerated or responded in a way that fluctuated across items [68]. A Z*h* value of < -2 indicates misfitting, where respondents typically select extreme responses, and a value of >  + 2 indicates overfitting, where respondents typically select the middle response: all values in between indicated well-fitting respondents [68]. Items with a high proportion of misfitting or overfitting were considered for removal.

Overall, decisions on whether items should be retained or removed were made with referral back to cognitive interview data [23] to ensure the voice of the general population was incorporated and appropriately reflected in the development of this questionnaire.

### Study 2

#### Study participants and recruitment

Participant sampling, recruitment, screening and demographics were collected as per study 1, with 500 participants recruited between the 10^th^ October 2021 and 17^th^ October 2021. Participants involved in study 1 could not participate in study 2.

#### Item pool

The 100-item pool used in this study was taken directly from the IRT analysis conducted in Study 1. The allocation of the 100-items to each domain and component can be seen in Column A, Appendix 2. All items had response options on 5-point likert scales as described in Study 1, and eight items were reverse scored.

#### Data collection

Participants were asked to complete the 20-min item pool. Progress tracking, questionnaire time completion and compensation were the same as per Study 1.

#### Data analysis

Reliability and validity data extraction and analysis were repeated as per Study 1.

### Study 3

#### Study participants and recruitment

All participants from Study 2 were approached to re-complete the food literacy questionnaire via the recruitment methods described for Study 1. The survey was closed to respondents once 250 participants were recruited. Participants were recruited [69] between the 1^st^ November 2021 and 4th November 2021, two weeks after the study 2 data collection period closed. Participants completed the same screening items and demographic questions as in Study 1.

#### Item pool

The item pool described in Study 2 was used in this study.

#### Data collection

Participants were asked to complete the 20-min item pool. Progress tracking, questionnaire time completion and compensation were the same as per Study 1.

#### Data analysis

Test–retest reliability was assessed using intraclass correlation coefficients (ICC) using a two-way mixed effect with absolute agreement. The average rater ICC value was reported, and the interpretation of ICC values was as per Ratner, 2009 [70].

### Calculation of food literacy scores

Raw scores, the sum of each response category in a set of items, are reported to inflate the extent of change reported by participants across administrations of a questionnaire due to the equal scoring of items. To address this, the Rasch method of scoring was conducted as it provides a more accurate measurement along the continuum of the underlying construct [71–73].

Raw score-to-measure tables were developed for each statistical component of the food literacy questionnaire. Raw scores represent the sum of participant responses to the likert scale (1–5) for the total number of items included within that component and correspond to a Rasch logit. These were calculated using Winsteps, version 5.2.4.0, following methods proposed by Linacre [74]. Rasch logits were transformed into “user friendly rescaling” such that all statistical components are assessed on a scale of 10–100 using methods proposed by Linacre [75]. Ten corresponds to the minimum possible statistical component score, while 100 corresponds to the maximum possible statistical component score.

## Ethics

This study was conducted according to the guidelines laid down in the Declaration of Helsinki and all procedures involving research study participants were approved by the University Human Research Ethics Committee (UHREC) at QUT, Approval Number: 2000000004. Written informed consent was obtained from all participants.

## Results

### Study 1

#### Participant characteristics

The survey was opened 931 times. Forty-five respondents were screened out due to age (5%) and 48 did not agree to participate (5%). Six respondents did not provide their postcode (1%) and 328 did not complete the questionnaire (35%). Overall, 504 participants provided full data and were included in the analysis. The demographic characteristics of participants are reported in Table 1 and are reflective of the Australian population with relation to gender, education and state of residence [76] and fairly representative for age and annual individual income.

#### Principal Component Analysis (PCA)

Of the 11 theoretical components of food literacy, the assumption of unidimensionality was met for components 1.2, 2.1, 2.3, 3.1, 4.3. The remaining six theoretical components were split into two (1.1, 2.2, 3.2, 4.1) or three (1.3, 4.2) statistical food literacy components. This resulted in a total of 19 statistical components which are nested within the theoretical components and domains food literacy (see Table 2). Seventy-one items were removed as they were low loading, cross loading or did not fit within a clear principal components structure. The division of each item under the theoretical and statistical components of food literacy can be seen in Appendix 1. The KMO’s were > 0.5 and Bartlett’s test was significant for all statistical components of food literacy. The total variance explained by the statistical components was > 50% for 15 of the 19 statistical components.

**Table 2:** Assessment of unidimensionality using PCA and the resulting theoretical and statistical food literacy components

**Theoretical domains, components and statistical components of food literacy**	**KMO**^a^	**Bartlett’s test**			**Total Variance Explained**	
		**Chi-square**	**df**	**p-value**^b^	**Total**	**% variance explained**
**Domain 1**						
**1.1**						
**1.1.1**	0.85	0916.83	10	< 0.001	3.03	61
**1.1.2**	0.65	0221.15	6	< 0.001	1.84	47
**1.2**	0.63	0423.23	6	< 0.001	2.09	52
**1.3**						
**1.3.1**	0.83	1095.28	10	< 0.001	3.14	63
**1.3.2**	0.79	0650.99	6	< 0.001	2.56	64
**1.3.3**	0.75	0527.09	10	< 0.001	2.45	49
**Domain 2**						
**2.1**	0.85	1140.37	15	< 0.001	3.43	57
**2.2**						
**2.2.1**	0.77	0635.45	10	< 0.001	2.64	53
**2.2.2**	0.86	1040.18	21	< 0.001	3.41	49
**2.3**	0.73	0612.55	3	< 0.001	2.28	76
**Domain 3**						
**3.1**	0.92	1830.14	28	< 0.001	4.59	57
**3.2**						
**3.2.1**	0.81	1044.70	15	< 0.001	3.21	54
**3.2.2**	0.63	0264.51	3	< 0.001	1.85	62
**Domain 4**						
**4.1**						
**4.1.1**	0.87	0974.10	15	< 0.001	3.26	54
**4.1.2**	0.53	0442.94	3	< 0.001	1.84	61
**4.2**						
**4.2.1**	0.95	3327.67	55	< 0.001	6.47	59
**4.2.2**	0.85	1011.36	10	< 0.001	3.15	63
**4.2.3**	0.67	0534.12	3	< 0.001	2.16	72
**4.3**	0.84	1046.45	21	< 0.001	3.38	48

^a^KMO > 0.5 = sampling adequacy

^b^*p*-value < 0.05 = suitability of data for PCA

### Item Response Theory (IRT)

#### Assessment of targeting

Across the 19 PCMs, 26 items were identified as having disordered thresholds and are shown in Column C, Appendix 2. Threshold 2 (t2) was the most frequently disordered (*n* = 24, 92%). To resolve this, ‘disagree’ and ‘neutral’ responses were combined. After this, all thresholds were ordered (see Column E–H, Appendix 2) and CPCs demonstrated acceptable patterns. Thresholds for the 100-item food literacy questionnaire ranged from -3.69 to + 6.11.

#### Assessment of responsiveness

Item locations ranged from < -0.01 to + 1.53 logits, within the recommended range of -2 to + 2 (see Column D, Appendix 2). Six of the 19 statistical components had items on the same logit level, with four of these reporting two items on the same logit (1.1.1, 1.2, 2.2.2, 4.1.1) and two reporting three items on the same logit (2.1, 4.2.1).

Floor effects were reported for 14 of the 19 statistical components of food literacy. These were between 0–3%, all below the upper limit of 15% (see Column K-L, Appendix 2). Ceiling effects were reported for all statistical components of food literacy (3–20%). All values were below 20%, with sub-component 3.2.1 at the upper limit. On average, 38 participants per sub-component of food literacy reported values that were outside of ± 1 logit from the lower and upper item threshold ranges; meaning that around 92% of participants gave responses consistent with expectations.

#### Assessment of reliability

The person separation reliability and item separation reliability for the statistical components of food literacy were high at > 0.99 for all components (see Column M, N, Appendix 2).

#### Assessment of validity

Outfit MSQs for the 100-item food literacy questionnaire ranged from 0.48 to 1.42 (see Column Q, Appendix 2). Ninety-nine items had MSQs between 0.5 to 1.5, while one item, (6_3.2) was just below the MSQ criteria, with an item fit of 0.48.

Person fit statistics are shown in Column P-R, Appendix 2. Between 11–41 participants had responses that were considered misfitting in the food literacy questionnaire, with sub-component 3.1 reporting the highest level of misfit (*n* = 41, 8%). Participant responses which overfit the food literacy questionnaire were only reported for component 4.2.1 (*n* = 14, 3%).

### Study 2

#### Participant characteristics

The survey was opened 830 times. Sixteen respondents were screened out due to age (2%) and 15 did not agree to participate (2%). Two respondents did not provide their postcode (0.2%) and 294 only partially completed the questionnaire (35%). Overall, 503 participants provided complete data and were included in the analysis. The demographic characteristics of participants are summarised under Study 2 in Table 1. There were minimal differences in participant demographics between study 1 and study 2 administrations.

#### Item Response Theory (IRT)

##### Assessment of reliability

As in Study 1, the person separation reliability and item separation reliability for the statistical components of food literacy were high at > 0.99 (see Column S-T, Appendix 2).

##### Assessment of validity

Outfit MSQs for the 100-item food literacy questionnaire ranged from 0.518 – 1.362 (see Column Z, Appendix 2). All items had MSQs between 0.5 to 1.5.

Person fit statistics are shown in Column AB-AD, Appendix 2. Between 12–45 participants had responses that were considered misfitting in the food literacy questionnaire, with sub-component 2.1 reporting the highest level of misfit (*n* = 45, 9%). As before, participant responses which overfit the food literacy questionnaire were only reported for component 4.2.1 (*n* = 23, 4%).

### Study 3

#### Participant characteristics

Of the 503 respondents from Study 2, 269 completed the food literacy questionnaire a second time and were included in the analysis. The demographic characteristics of participants are summarised in Table 1. There were no important differences in demographics between study 2 and study 3 participants.

#### Test–retest reliability

The intraclass correlation coefficients for the statistical components of food literacy are reported in Column AE, Appendix 2. Moderate reliability was reported for five statistical components of food literacy (1.1.2, 2.1, 2.3, 4.1.2, 4.2.2), while good reliability was reported for the remaining 14 statistical components.

#### Calculation of food literacy scores

The raw score-to-measure table can be seen in Appendix 3. All possible raw scores for each statistical component are listed in Column B, Rasch logits are listed in Column C and the user-friendly re-scaled scores are in Column D.

#### How to score the IFLQ-19

The International Food Literacy Questionnaire reflects the 19 statistical components of food literacy and thus, is called the IFLQ-19. The methods for scoring are seen in Table 3 below. The IFLQ-19 produces 19 separate scores for each of the statistical components.

**Table 3:** How to score each component of the IFLQ-19

**How to score each component of the IFLQ-19**
1) **Identify the raw score for each item:** The final IFLQ-19 can be seen in Table 4. The response categories for each item are listed in the second column (Response categories (score)) and each response category is allocated a value. For example, Q1_1.1.1 has five response categories, where strongly disagree is valued at 1, disagree is valued at 2, neutral valued at 3, agree valued at 4 and strongly agree valued at 5
2) **Sum the raw scores for items under each statistical component:** All items listed under the statistical component headings need to be scored, where the response category to each item needs to be totaled. For example, in statistical component 1.1.1, items 1_1.1.1 through to 5_1.1.1 will be summed where a total of 5 to 23 can be achieved. This is the raw score
3) **Determine the re-scaled score:** Download the Appendix 3 document, and under column B, find the raw score for the statistical component achieved by the respondent to obtain their re-scaled score. For example, if an individual obtained a raw score of 5 (Column B, Row 4), the re-scaled score would be 10. Please note, raw scores and re-scaled scores are different for each statistical component
4) **Interpreting the re-scaled score:** a) If administered at a single time point, the rescaled score can be used to rank individuals on their level of food literacy for a given statistical component. It can also be used to compare respondents. For example, respondent one’s rescaled score of 10 (Column D, Row 4) compared to respondent two’s rescaled score of 43 (Column D, Row 13) suggests respondent two has a higher level of the latent trait (the statistical component of food literacy) compared to respondent one b) If administered across multiple time points, it can be used to represent an individual’s progress toward the maximum possible score on the statistical component, which provides a more accurate representation of the change. For example, if respondent one’s rescaled score changed from 10 (Column D, Row 4) to 38 (Column D, Row 9), this indicates a four-fold increase in the statistical component of food literacy c) Rescaled scores can also be compared to other variables. For example, a correlation with participant socio-demographics and statistical components of food literacy
5) **For consideration:** Further research is required to determine whether a score on one statistical component represents an equivalent score on another. In its current form, the IFLQ-19 is not designed for rescaled statistical component scores to be summed to obtain a score at the theoretical component, domain or total food literacy level

**Table 4:** The food literacy questionnaire (IFLQ-19)

Domains, Components, Statistical components and items	Response categories (score)
**Domain 1: Plan and Manage**	
**Component 1.1 Prioritise time and money for food**	
**1.1.1**	
Q1_1.1.1R: Compared to other daily activities, food shopping takes up too much of my time	a.Strongly disagree (1) b.Disagree (2) c.Neutral (3) d.Agree (4) e.Strongly agree (5)
Q2_1.1.1R: Compared to other daily activities, cooking or preparing food takes up too much of my time	a.Strongly disagree (1) b.Disagree (2) c.Neutral (3) d.Agree (4) e.Strongly agree (5)
Q3_1.1.1R: I often run out of time to buy or prepare the food I'd prefer	a.Strongly disagree (1) b.Disagree (2) c.Neutral (3) d.Agree (3) e.Strongly agree (4)
Q4_1.1.1R: Compared to other daily activities, eating takes up too much of my time	a.Strongly disagree (1) b.Disagree (2) c.Neutral (3) d.Agree (3) e.Strongly agree (4)
Q5_1.1.1R: Compared to other daily expenses, food takes up too much of my budget	a.Strongly disagree (1) b.Disagree (2) c.Neutral (3) d.Agree (4) e.Strongly agree (5)
**1.1.2**	
Q1_1.1.2: I always try to have enough money set aside to feed myself and/or the people I'm responsible for	a.Strongly disagree (1) b.Disagree (2) c.Neutral (2) d.Agree (3) e.Strongly agree (4)
Q2_1.1.2: When I'm running low on money, I prioritise foods based on health	a.Strongly disagree (1) b.Disagree (2) c.Neutral (3) d.Agree (4) e.Strongly agree (5)
Q3_1.1.2: When I'm running low on money, I prioritise foods based on taste	a.Strongly disagree (1) b.Disagree (2) c.Neutral (3) d.Agree (4) e.Strongly agree (5)
Q4_1.1.2: When I'm running low on money, I prioritise foods based on cost	a.Strongly disagree (1) b.Disagree (2) c.Neutral (3) d.Agree (4) e.Strongly agree (5)
**Component 1.2 Plan food intake (formally and informally) so that food can be regularly accessed through some source, irrespective of changes in circumstances or environment**	
Q1_1.2: I plan ahead (e.g. for the day or week) when preparing my own food	a.Never (1) b.Rarely (2) c.Sometimes (3) d.Often (4) e.Always (5)
Q2_1.2: I plan what to buy before I go food shopping	a.Never (1) b.Rarely (2) c.Sometimes (2) d.Often (3) e.Always (4)
Q3_1.2: I am able to adapt my plans for what to eat even if my circumstances change	a.Strongly disagree (1) b.Disagree (2) c.Neutral (3) d.Agree (4) e.Strongly agree (5)
Q4_1.2: I am able to adapt my plans for what to eat even if the food I plan to eat isn't available	a.Strongly disagree (1) b.Disagree (2) c.Neutral (3) d.Agree (4) e.Strongly agree (5)
**Component 1.3 Make feasible food decisions which balance food needs (e.g. nutrition, taste, hunger) with available resources (e.g. time, money, skills, equipment)**	
**1.3.1**	
Q1_1.3.1: When buying from a restaurant, cafe or takeaway, I know how much money I spend in an average week	a.Strongly disagree (1) b.Disagree (2) c.Neutral (3) d.Agree (4) e.Strongly agree (5)
Q2_1.3.1: When buying from a restaurant, cafe or takeaway, I compare prices before I buy food	a.Strongly disagree (1) b.Disagree (2) c.Neutral (3) d.Agree (4) e.Strongly agree (5)
Q3_1.3.1: When buying from a restaurant, cafe or takeaway, I try to get the best food for the best price	a.Strongly disagree (1) b.Disagree (2) c.Neutral (3) d.Agree (4) e.Strongly agree (5)
Q4_1.3.1: When buying from a restaurant, cafe or takeaway, I compare prices between similar products in order to get the best value	a.Strongly disagree (1) b.Disagree (2) c.Neutral (3) d.Agree (4) e.Strongly agree (5)
Q5_1.3.1: When buying from a restaurant, cafe or takeaway, I plan to take advantage of promotions	a.Strongly disagree (1) b.Disagree (2) c.Neutral (3) d.Agree (4) e.Strongly agree (5)
**1.3.2**	
Q1_1.3.2: When food shopping, I compare prices before I buy food	a.Strongly disagree (1) b.Disagree (2) c.Neutral (3) d.Agree (4) e.Strongly agree (5)
Q2_1.3.2: When food shopping, I try to get the best food for the best price	a.Strongly disagree (1) b.Disagree (2) c.Neutral (3) d.Agree (4) e.Strongly agree (5)
Q3_1.3.2: When food shopping, I compare prices between similar products in order to get the best value	a.Strongly disagree (1) b.Disagree (2) c.Neutral (3) d.Agree (4) e.Strongly agree (5)
Q4_1.3.2: When food shopping, I plan to take advantage of promotions	a.Strongly disagree (1) b.Disagree (2) c.Neutral (3) d.Agree (4) e.Strongly agree (5)
**1.3.3**	
Q1_1.3.3: When food shopping, I know how much money I spend in an average week	a.Strongly disagree (1) b.Disagree (2) c.Neutral (3) d.Agree (4) e.Strongly agree (5)
Q2_1.3.3: Even if I don't have my normal amount of time, I can still et the food I prefer	a.Strongly disagree (1) b.Disagree (2) c.Neutral (3) d.Agree (4) e.Strongly agree (5)
Q3_1.3.3: Even if I don't have my normal kitchen equipment, I can still eat the food I prefer	a.Strongly disagree (1) b.Disagree (2) c.Neutral (3) d.Agree (4) e.Strongly agree (5)
Q4_1.3.3: Even if I'm hungry, I prepare the food I had planned	a.Strongly disagree (1) b.Disagree (2) c.Neutral (3) d.Agree (4) e.Strongly agree (5)
Q5_1.3.3: I balance my nutritional needs with the time, money, skills and kitchen equipment I have	a.Strongly disagree (1) b.Disagree (2) c.Neutral (2) d.Agree (3) e.Strongly agree (4)
**Domain 2: Select**	
**Component 2.1 Access food through multiple sources and know the advantages and disadvantages of these**	
Q1_2.1: When eating out, it's important to me that I can find the food I prefer	a.Strongly disagree (1) b.Disagree (2) c.Neutral (2) d.Agree (3) e.Strongly agree (4)
Q2_2.1: When food shopping in a familiar place, I find the foods I prefer to eat	a.Strongly disagree (1) b.Disagree (2) c.Neutral (2) d.Agree (3) e.Strongly agree (4)
Q3_2.1: When food shopping in a new place, I find the foods I prefer to eat	a.Strongly disagree (1) b.Disagree (2) c.Neutral (3) d.Agree (4) e.Strongly agree (5)
Q4_2.1: When buying food from a familiar restaurant, cafe or takeaway, I find the foods I prefer to eat	a.Strongly disagree (1) b.Disagree (2) c.Neutral (2) d.Agree (3) e.Strongly agree (4)
Q5_2.1: When buying food from a new restaurant, cafe or takeaway, I find the foods I prefer to eat	a.Strongly disagree (1) b.Disagree (2) c.Neutral (2) d.Agree (3) e.Strongly agree (4)
Q6_2.1: I find the foods I can afford	a.Strongly disagree (1) b.Disagree (2) c.Neutral (3) d.Agree (4) e.Strongly agree (5)
**Component 2.2 Determine what is in a food product, where it came from, how to store it and use it**	
**2.2.1**	
Q1_2.2.1: I know how to find information on how fresh food is grown and produced	a.Strongly disagree (1) b.Disagree (2) c.Neutral (3) d.Agree (4) e.Strongly agree (5)
Q2_2.2.1: The ingredients in packaged food products is important to me when deciding what foods to buy	a.Not at all important (1) b.Slightly important (2) c.Neutral (3) d.Very important (4) e.Extremely important (5)
Q3_2.2.1: I compare either the kilojoules, fat, sugar or salt content on food products to guide what I buy	a.Strongly disagree (1) b.Disagree (2) c.Neutral (2) d.Agree (3) e.Strongly agree (4)
Q4_2.2.1: I know where to find information on the environmental and ethical impact of different foods	a.Strongly disagree (1) b.Disagree (2) c.Neutral (3) d.Agree (4) e.Strongly agree (5)
Q5_2.2.1: When food shopping, I know how my food is stored before I purchase it	a.Strongly disagree (1) b.Disagree (2) c.Neutral (3) d.Agree (4) e.Strongly agree (5)
**2.2.2**	
Q1_2.2.2: I find it easy to know what country different foods come from	a.Strongly disagree (1) b.Disagree (2) c.Neutral (3) d.Agree (4) e.Strongly agree (5)
Q2_2.2.2: I try to buy fresh food that is currently in season in my country	a.Strongly disagree (1) b.Disagree (2) c.Neutral (2) d.Agree (3) e.Strongly agree (4)
Q3_2.2.2: When eating out, I can make a judgement on the ingredients in the food I've selected	a.Strongly disagree (1) b.Disagree (2) c.Neutral (3) d.Agree (4) e.Strongly agree (5)
Q4_2.2.2: When eating out, I can make a judgement on the nutritional value of the food I've selected	a.Strongly disagree (1) b.Disagree (2) c.Neutral (3) d.Agree (4) e.Strongly agree (5)
Q5_2.2.2: I know where to look for information on what's in packaged foods	a.Strongly disagree (1) b.Disagree (2) c.Neutral (2) d.Agree (3) e.Strongly agree (4)
Q6_2.2.2: When food shopping, I know what's in packaged foods that I could buy	a.Strongly disagree (1) b.Disagree (2) c.Neutral (3) d.Agree (4) e.Strongly agree (5)
Q7_2.2.2: I know how to store fruits and vegetables for best freshness and food safety	a.Strongly disagree (1) b.Disagree (2) c.Neutral (3) d.Agree (4) e.Strongly agree (5)
**Component 2.3 Judge the quality of food**	
Q1_2.3R: I am disappointed with my selection of fresh food because it doesn't meet my expectations	a.Never (1) b.Rarely (2) c.Sometimes (3) d.Often (4) e.Always (5)
Q2_2.3R: I am disappointed with my selection of processed or convenience food because it doesn't meet my expectations	a.Never (1) b.Rarely (2) c.Sometimes (3) d.Often (4) e.Always (5)
Q3_2.3R: I am disappointed with my selection of foods when eating out because it doesn't meet my expectations	a.Never (1) b.Rarely (2) c.Sometimes (3) d.Often (4) e.Always (5)
**Domain 3: Prepare**	
**Component 3.1 Make a good tasting meal form whatever is available. This includes being able to prepare commonly available foods, efficiently use common pieces of kitchen equipment, and having sufficient repertoire of skills to adapt recipes (written or unwritten) to experiment with food and ingredients**	
Q1_3.1: I am able to prepare and eat the food I prefer even if something unexpected happens in the short term	a.Strongly disagree (1) b.Disagree (2) c.Neutral (3) d.Agree (4) e.Strongly agree (5)
Q2_3.1: I have the skills to prepare and cook affordable foods that I prefer	a.Strongly disagree (1) b.Disagree (2) c.Neutral (3) d.Agree (4) e.Strongly agree (5)
Q3_3.1: I can prepare a meal using fresh or minimally processed ingredients	a.Strongly disagree (1) b.Disagree (2) c.Neutral (2) d.Agree (3) e.Strongly agree (4)
Q4_3.1: When preparing food, I am confident about substituting alternative ingredients	a.Strongly disagree (1) b.Disagree (2) c.Neutral (3) d.Agree (4) e.Strongly agree (5)
Q5_3.1: I am able to prepare the food I prefer even if my health condition changes	a.Strongly disagree (1) b.Disagree (2) c.Neutral (3) d.Agree (4) e.Strongly agree (5)
Q6_3.1: I know how to find information about preparing different foods	a.Strongly disagree (1) b.Disagree (2) c.Neutral (3) d.Agree (4) e.Strongly agree (5)
Q7_3.1: When preparing food, I know what to do when something goes wrong	a.Strongly disagree (1) b.Disagree (2) c.Neutral (3) d.Agree (4) e.Strongly agree (5)
Q8_3.1: I am confident preparing food from the ingredients I have on hand	a.Not at all confident (1) b.Slightly confident (2) c.Neutral (3) d.Moderately confident (4) e.Extremely confident (5)
**Apply basic principles of safe food hygiene and handling**	
**3.2.1**	
Q1_3.2.1: I wash fruit and vegetables before eating them	a.Never (1) b.Rarely (2) c.Sometimes (3) d.Often (4) e.Always (5)
Q2_3.2.1: After handling raw meat, poultry or fish, I wash my hands	a.Never (1) b.Rarely (2) c.Sometimes (3) d.Often (4) e.Always (5)
Q3_3.2.1: After slicing raw meat, poultry or fish, I set the cutting board aside and use a different cutting board for other foods	a.Never (1) b.Rarely (2) c.Sometimes (2) d.Often (3) e.Always (3)
Q4_3.2.1: Before handling food, I always wash my hands	a.Never (1) b.Rarely (2) c.Sometimes (3) d.Often (4) e.Always (5)
Q5_3.2.1: I read the storage and expiry date information on packaged food	a.Never (1) b.Rarely (2) c.Sometimes (3) d.Often (4) e.Always (5)
Q6_3.2.1: I use the storage and expiry date information on food when deciding whether to eat it	a.Never (1) b.Rarely (2) c.Sometimes (3) d.Often (4) e.Always (5)
**3.2.2**	
Q1_3.2.2: To prevent food poisoning, your freezer temperature should be at or below -18 degrees Celsius	a.Not at all sure (1) b.Somewhat sure (2) c.Neutral (2) d.Moderately sure (3) e.Very sure (4)
Q2_3.2.2: To prevent food poisoning, your refrigerator temperature should be at or below 4 degrees Celsius	a.Not at all sure (1) b.Somewhat sure (2) c.Neutral (3) d.Moderately sure (4) e.Very sure (5)
Q3_3.2.2: Micro-organisms that cause food poisoning grow in temperatures between 5–60 degrees Celsius	a.Strongly disagree (1) b.Disagree (2) c.Neutral (2) d.Agree (3) e.Strongly agree (4)
**Domain 4: Eat**	
**Component 4.1 Understand food has an impact on personal wellbeing**	
**4.1.1**	
Q1_4.1.1: I know what foods to eat to keep me healthy	a.Strongly disagree (1) b.Disagree (2) c.Neutral (2) d.Agree (3) e.Strongly agree (4)
Q2_4.1.1: Eating more fruits and vegetables lowers your risk of heart disease	a.Strongly disagree (1) b.Disagree (2) c.Neutral (2) d.Agree (3) e.Strongly agree (4)
Q3_4.1.1: Eating foods high in saturated fat increases your risk of cardiovascular disease	a.Strongly disagree (1) b.Disagree (2) c.Neutral (2) d.Agree (3) e.Strongly agree (4)
Q4_4.1.1: Eating foods high in sugar increases your risk of tooth decay	a.Strongly disagree (1) b.Disagree (2) c.Neutral (3) d.Agree (4) e.Strongly agree (5)
Q5_4.1.1: Eating foods high in salt increases your risk of high blood pressure	a.Strongly disagree (1) b.Disagree (2) c.Neutral (2) d.Agree (3) e.Strongly agree (4)
Q6_4.1.1: Eating more milk, yoghurt and cheese decreases your risk of weak bones	a.Strongly disagree (1) b.Disagree (2) c.Neutral (3) d.Agree (4) e.Strongly agree (5)
**4.1.2**	
Q1_4.1.2: The type of food I eat influences my health	a.Strongly disagree (1) b.Disagree (2) c.Neutral (3) d.Agree (4) e.Strongly agree (5)
Q2_4.1.2: The type of food I eat influences my wellbeing	a.Strongly disagree (1) b.Disagree (2) c.Neutral (2) d.Agree (3) e.Strongly agree (4)
Q3_4.1.2: My emotions influence my food choices	a.Never (1) b.Rarely (2) c.Sometimes (3) d.Often (4) e.Always (5)
**Component 4.2 Demonstrate self-awareness of the need to personally balance food intake. This influences knowing foods to include for good health, foods to restrict for good health and appropriate portion size and frequency**	
**4.2.1**	
Q1_4.2.1: The Australian Dietary Guidelines recommend that people should eat vegetables every day	a.Not at all sure (1) b.Somewhat sure (2) c.Neutral (3) d.Moderately sure (4) e.Very sure (5)
Q2_4.2.1: The Australian Dietary Guidelines recommend that people should eat fruit every day	a.Not at all sure (1) b.Somewhat sure (2) c.Neutral (3) d.Moderately sure (4) e.Very sure (5)
Q3_4.2.1: The Australian Dietary Guidelines recommend that people should limit sugary foods and drinks	a.Not at all sure (1) b.Somewhat sure (2) c.Neutral (3) d.Moderately sure (4) e.Very sure (5)
Q4_4.2.1: The Australian Dietary Guidelines recommend that people should eat wholegrains every day	a.Not at all sure (1) b.Somewhat sure (2) c.Neutral (3) d.Moderately sure (4) e.Very sure (5)
Q5_4.2.1: The Australian Dietary Guidelines recommend that people should drink water every day	a.Not at all sure (1) b.Somewhat sure (2) c.Neutral (3) d.Moderately sure (4) e.Very sure (5)
Q6_4.2.1: The Australian Dietary Guidelines recommend that people should limit processed meats	a.Not at all sure (1) b.Somewhat sure (2) c.Neutral (3) d.Moderately sure (4) e.Very sure (5)
Q7_4.2.1: The Australian Dietary Guidelines recommend that people should limit foods with saturated fats	a.Not at all sure (1) b.Somewhat sure (2) c.Neutral (3) d.Moderately sure (4) e.Very sure (5)
Q8_4.2.1: The Australian Dietary Guidelines recommend that people should eat milk, yoghurt, cheese and alternatives every day	a.Not at all sure (1) b.Somewhat sure (2) c.Neutral (2) d.Moderately sure (3) e.Very sure (4)
Q9_4.2.1: The Australian Dietary Guidelines recommend that people should limit foods with added salt	a.Not at all sure (1) b.Somewhat sure (2) c.Neutral (3) d.Moderately sure (4) e.Very sure (5)
Q10_4.2.1: The Australian Guide to Healthy Eating recommends that adults should eat around 5 serves of vegetables and legumes/beans per day	a.Not at all sure (1) b.Somewhat sure (2) c.Neutral (3) d.Moderately sure (4) e.Very sure (5)
Q11_4.2.1: The Australian Guide to Healthy Eating recommends that adults should eat around 2 serves of fruit per day	a.Not at all sure (1) b.Somewhat sure (2) c.Neutral (3) d.Moderately sure (4) e.Very sure (5)
**4.2.2**	
Q1_4.2.2: A serve of vegetables is ½ medium potato	a.Not at all sure (1) b.Somewhat sure (2) c.Neutral (3) d.Moderately sure (4) e.Very sure (5)
Q2_4.2.2: A serve of fruit is 4 dried apricots	a.Not at all sure (1) b.Somewhat sure (2) c.Neutral (3) d.Moderately sure (4) e.Very sure (5)
Q3_4.2.2: A serve of grains (cereal) foods is ½ cup cooked porridge	a.Not at all sure (1) b.Somewhat sure (2) c.Neutral (3) d.Moderately sure (4) e.Very sure (5)
Q4_4.2.2: A serve of lean meats, poultry, fish, eggs, tofu, nuts and seeds is 1 cup baked beans	a.Not at all sure (1) b.Somewhat sure (2) c.Neutral (2) d.Moderately sure (3) e.Very sure (4)
Q5_4.2.2: A serve of milk, yoghurt, cheese and/or alternatives is 2 slices of cheese	a.Not at all sure (1) b.Somewhat sure (2) c.Neutral (3) d.Moderately sure (4) e.Very sure (5)
**4.2.3**	
Q1_4.2.3: I make a conscious effort to try and eat healthily	a.Strongly disagree (1) b.Disagree (2) c.Neutral (3) d.Agree (4) e.Strongly agree (5)
Q2_4.2.3: When deciding what to eat, I think about healthy choices	a.Strongly disagree (1) b.Disagree (2) c.Neutral (3) d.Agree (4) e.Strongly agree (5)
Q3_4.2.3: I use the nutritional label on packaged food products to guide my purchases	a.Strongly disagree (1) b.Disagree (2) c.Neutral (3) d.Agree (4) e.Strongly agree (5)
**Component 4.3 Join in and eat in a social way**	
Q1_4.3: I am comfortable eating with other people	a.Strongly disagree (1) b.Disagree (2) c.Neutral (3) d.Agree (4) e.Strongly agree (5)
Q2_4.3: Eating with other people is about more than just food	a.Strongly disagree (1) b.Disagree (2) c.Neutral (3) d.Agree (4) e.Strongly agree (5)
Q3_4.3: Eating brings people together in an enjoyable way	a.Strongly disagree (1) b.Disagree (2) c.Neutral (2) d.Agree (3) e.Strongly agree (4)
Q4_4.3: When eating with other people, it is important to me to sit down and eat at a table	a.Not at all important (1) b.Slightly important (2) c.Neutral (2) d.Moderately important (3) e.Extremely important (4)
Q5_4.3: I eat together with other people	a.Never (1) b.Rarely (2) c.Sometimes (3) d.Often (4) e.Always (5)
Q6_4.3: Food is a central part of how I make friends or form relationships with other people	a.Strongly disagree (1) b.Disagree (2) c.Neutral (3) d.Agree (4) e.Strongly agree (5)
Q7_4.3: Food is a key part of how I celebrate occasions or cultural events with other people	a.Strongly disagree (1) b.Disagree (2) c.Neutral (2) d.Agree (3) e.Strongly agree (4)

## Discussion

This study described the methods used to develop a 100-item food literacy questionnaire, the IFLQ-19. This questionnaire comprehensively reflects the 11 theoretical components of food literacy by Vidgen & Gallegos [1], which gives valid, reliable and consistent results.

### PCA

The principal components analysis was integral in determining the structure of the food literacy questionnaire. While the components of food literacy were theorised to be unidimensional, findings from this research rejected this theory in six cases. Components which had more comprehensive descriptions generally addressing multiple interrelated points (see Fig. 1) were often split, for example component 1.1, ‘Prioritise money and time for food’ was split into sub-component 1.1.1 addressing the ‘time’ aspect, while sub-component 1.1.2 addressed the context of ‘food’. The 19 statistical components resulting from the PCA were retained for the following reasons: 1) This research prioritised the voice of the general population obtained through cognitive interviews [23]. 2) Comprehensive measurement of the theoretical components required the statistical components to be retained. 3) Interventions targeting different aspects of food literacy can choose the appropriate statistical component to use in their evaluation. While it is theorised that the statistical components sit within the 11 theoretical components proposed by Vidgen & Gallegos, further statistical testing using a Multidimensional PCM (MPCM) is needed to verify this structure. The total variance explained was between 46–49% for four of the statistical components (1.1.2, 1.3.3, 2.2.2, 4.3). As this was just below the threshold (< 50%) and to ensure Vidgen & Gallegos [1] model was comprehensively addressed, these four statistical components were retained.

### Targeting and responsiveness

Targeting and responsiveness were assessed using item thresholds, category probability curves, item locations and person-item maps. In the initial analysis, 26 item thresholds were not correctly ordered. The t2 category was most often disordered, meaning respondents were unable to reliably discriminate between the ‘disagree’ and ‘neutral’ categories. This can occur for two reasons: 1) there were too many response categories, and the ‘neutral’ option should be removed, and 2) there may be unequal category responses [56]. While the former is more difficult to confirm, the latter was identified in this situation, where respondents tended to select the upper categories (often/always, agree/strongly agree) compared to the lower options across the 26-items. The scale chosen for items, such as ‘strongly disagree’ to ‘strongly agree’ instead of ‘never’ to ‘always’ was also considered as a potential issue in the selection of response categories. However, this was not identified in cognitive interviews with participants on these items [23], suggesting that if there was an impact, it would have been minimal. Overall, disordered thresholds were low (26%) and category combining at the analysis phase resulted in ordered thresholds for all items; thus, response categories were working as intended and respondents were distributed across lower to higher food literacy levels [77]. No items were deleted, as they were all considered to be critical in assessing the statistical component of food literacy.

Item locations were all acceptable, though tended to cluster in the mid to higher end of the recommended range, from + 0 to + 1.5 logits. This suggests that while item locations were adequate, additional items that more equally spread across the -2 to + 2 range would have ensured differentiation between participants and their food literacy characteristics could be better captured [78]. Multiple items sat on the same logit across six statistical components, suggesting that items were assessing the same level of food literacy. However, all items were retained due to previous qualitative feedback [23] on ensuring the components of food literacy were comprehensively addressed.

Person-item maps identified that fewer respondents obtained the minimum possible score, meaning floor effects were negligible [79]. In contrast, at least 13 respondents obtained the highest possible score for each sub-section of the food literacy questionnaire indicating mild ceiling effects [79]. This suggests there may be a slight bias toward respondents with higher food literacy levels [80]. However, all values were below the upper limit for floor and ceiling effects, suggesting the food literacy questionnaire is an appropriate measurement model [81]. Overall, targeting was acceptable and responsiveness of the food literacy questionnaire was supported.

### Validity

Validity was assessed using MSQs from item fit analysis and Zh statistics from person-fit analysis. In study 1, only one item had an MSQ value just below the 0.5 to 1.5 recommended criteria. While items with MSQs < 0.5 are considered to be less productive in measurement, considering item 6_3.2 reported an MSQ of 0.48, the impact on the overall questionnaire would have been negligible. In study 2, all MSQ values were within the 0.5 to 1.5 range. This suggests that the food literacy questionnaire measures what was intended, and that all items work well together and are appropriate for combined scoring [39].

With relation to person-fit, Z-statistics were used to determine misfitting and overfitting respondents. In study 1 and 2, respondents classified as ‘overfitting’ were only reported for sub-component 4.2.1, where respondents across this section predominantly selected 2, ‘somewhat sure’ and 3 ‘neutral’. All items in this section related to knowing food group recommendations from the Australian Dietary Guidelines [82] and Australian Guide to Healthy Eating [83]. If someone was unable to answer the first item, it was unlikely they responded in the extremes for similar items, thus explaining the higher number of ‘neutral’ responses for items in this sub-section. In study 1 there were between 11–41 and in study 2 between 12–45 respondents who were considered ‘misfitting’. A review of participants who were classified as misfitting, identified they tended to select 5, ‘strongly agree/always’ and 4 ‘agree/often’ responses rather than those at the lower end of the scale. This is unsurprising, considering findings from the ‘targeting and responsiveness’ statistics which suggest there was a mild ceiling effect in the questionnaire. Further, misfit differences across administrations is common due to latent trait distribution and item parameter drift [84, 85]. While misfitting and overfitting items may impact the validity of a questionnaire, this does not appear to be the case here [86]. Overall, validity for the food literacy questionnaire was high.

### Reliability

Person separation and item separation reliability were very high in study 1 and 2 for all statistical components of food literacy. This suggests that the questionnaire was sensitive in distinguishing between low and high performers and that the sample was large enough to locate items on the latent variables [39, 52]. The ICCs for the statistical components were moderate to good, though none reported excellent ICCs. This may be due to some variability in participants circumstances (e.g. they may not run low on money or shop/eat at new restaurants) or interpretation on types of foods. Further, it may also be a result of the mild ceiling effects described above, whereby a less equal distribution of respondent scores has been reported to be associated with lower ICCs [87]. However, no statistical components reported ‘poor’ ICCs and thus, the questionnaire is still considered to have a good level of reliability.

### Scoring the IFLQ-19

Re-scaled scoring was chosen as raw scores (grounded in CTT), assumes the distance between response options is the same. However, IRT methods, and in particular, our research found this was not the case. Therefore, re-scaled scoring provided a more precise indication of a person’s level on the statistical food literacy components and can be used to assess magnitude of change between questionnaire administrations and the relationship between other variables. However, this does increase the complexity of scoring. In acknowledgement of this, the authors developed a scoring guide for future users of the tool. This conversion table (Appendix 3) means practitioners do not need to conduct Rasch analysis every time to obtain Rasch transformed score and can refer to this table for ease of scoring.

## Strengths and limitations

The strengths of this research include the rigorous and comprehensive development of the questionnaire and adherence to the Vidgen & Gallegos model [1]. The IFLQ-19 developed in this study is the result of several comprehensive, multi-step research studies over the course of several years. This included international consultation and feedback on the initial pool of food literacy items which was reviewed with general public and validated in a diverse sample of Australian adults [3, 23]. The international perspective and the use of IRT as a statistical method is a distinct advantage over previous approaches [8–19]. As IRT is sample-independent [88, 89], the questionnaire validated from this study can be re-administered in international populations. With previous international consultation [3] forming the basis of items within this questionnaire, we expect the IFLQ-19 to hold relevance across Western and middle to high income countries, with statistical testing now feasible across a broad range of cultures and economic levels internationally. This research favoured the perspective of the general population as obtained through early cognitive interview studies [23]. As the Vidgen & Gallegos conceptualisation [1] was designed with the general population to determine knowledge, skills and behaviours that encompass food literacy, it was integral the final questionnaire reflected this perspective over statistical cut-offs. Existing food literacy surveys have been limited due to their use of varying definitions or low adherence to the Vidgen & Gallegos conceptual [1] of food literacy. Recent research identified the Vidgen & Gallegos [1] model as the core conceptualisation of food literacy [4], thus, the development of a questionnaire which comprehensively addresses this framework was needed [4]. Finally, this food literacy questionnaire fills a substantial gap in that food literacy, a consumer behaviour, has been difficult to conceptualise and measure [90]. Thus, food systems monitoring and surveillance to date has been limited in assessing this construct [90]. This questionnaire aims to fill this gap by measuring the key components of acquisiting, preparation, meal practices and storage [90]. This allows for further investigation of the relationship between food literacy and the broader food system, including the impact on retail, marketing, food environments [90], dietary intake and food security [2].

The limitations of this research include the collapse of disordered categories during the IRT analysis and the length of the IFLQ-19. While the collapsing of response categories in Study 1 addressed threshold disordering, the questionnaire was not administered in Study 2 with the re-formed categories. As threshold ordering is based on an a priori structure, empirical review is recommended [55]. However, this was not conducted for pragmatic reasons, as forced choice, where too few categories are provided or missing intervals in response choices can cause respondents confusion, resulting in imprecise category choices [91–93].

The final IFLQ-19 developed from this research consisted of 100-items which is expected to take approximately 20-min to complete. In order to reduce participant burden, methods such as Computerised Adaptive Testing (CAT), where respondents receive a unique set of items from a larger item bank based on their responses, are recommended [49].

Future research should explore the use of CAT for the IFLQ-19, conduct the relevant statistics so the IFLQ-19 can be scored at the theoretical component, domain and overall level and examine associations with diet quality and food security to determine if higher scores on statistical components of food literacy are meaningfully associated with these constructs. Further, resources and videos will be available online or presented as part of workshops and at conferences on the use and scoring of the IFLQ-19 to assist with policy and practice implementation.

## Conclusion

This study progressed the development of a comprehensive, validated food literacy questionnaire using item response theory. The resulting 100-item food literacy questionnaire reflected the 4 domains of planning and managing (6 statistical components), selecting (4 statistical components), preparing (3 statistical components) and eating (6 statistical components). The food literacy questionnaire had acceptable and supported targeting and responsiveness, high validity and good reliability in a diverse sample of Australian adults. This questionnaire fills a substantial gap in the conceptualisation, measurement, monitoring and surveillance of food literacy, a consumer behaviour, internationally: a key aspect within the broader food system.

## Supplementary Information

**Additional file 1.**

## Data Availability

The datasets used and/or analysed during the current study are available from the corresponding author on reasonable request.

## Abbreviations

CPCs: Category Probability Curves
CTT: Classical Test Thoery
ICC: Intraclass Correlation Coefficients
IFLQ-19: International Food Literacy Questionnaire -19
IRT: Item Response Theory
KMO: Kaiser-Meyer Olkin
MPCM: Multidimensional Partial Credit Model
MSQs: Outfit Mean-Squares
PCA: Principal Component Analysis
PCM: Partial Credit Model
